# Effects of nitrogen reduction combined with bio-organic fertilizer on soil bacterial community diversity of red raspberry orchard

**DOI:** 10.1371/journal.pone.0283718

**Published:** 2023-07-11

**Authors:** Xu Yuan, Jiaan Zhang, Feiyang Chang, Xinyue Wang, Xuemei Zhang, Haoan Luan, Guohui Qi, Suping Guo

**Affiliations:** Institute of Forestry, Hebei Agricultural University, Baoding, Hebei Province, China; University of Salento Department of Biological and Environmental Sciences and Technologies: Universita del Salento Dipartimento di Scienze e Tecnologie Biologiche ed Ambientali, ITALY

## Abstract

Understanding soil bacterial diversity under nitrogen reduction is necessary for the crucial role in soil nitrogen cycling. However, the effects of combined fertilization on soil chemical properties, microbial community structure, and yield are unknown. This study was conducted to investigate the effect of nitrogen fertilizer reduction with bio-organic fertilizer on soil bacterial community diversity of red raspberry orchard. Six treatments were set in this study: NF-100%, NF-75%, NF-50%, NF-25% and CF, no nitrogen fertilizer and bio-organic fertilizer for CK. The bacterial community structures of soil were analyzed by 16S rRNA gene amplification high-throughput sequencing technology. Nitrogen fertilizer reduction with bio-organic fertilizer increased soil organic matter (SOM), total nitrogen (TN), alkali-hydrolyzable nitrogen (AN), available phosphorus (AP), available potassium (AK), and reduced soil pH. NF-50% and NF-25% treatments increased the yield of red raspberry. Nitrogen reduction combined with bio-organic fertilizer increased the relative abundance of copiotrophic bacteria and decreased the relative abundance of oligotrophic bacteria. The increase in copiotrophic bacteria in the soil of red raspberry orchard could indicate an increase in soil nutrient availability, which have positive implications for soil fertility and production. However, nitrogen fertilizer reduction with bio-organic fertilizer altered the abundance and diversity of soil bacteria, which was reduced compared to CF treatments. The PCoA analysis of the soil bacterial community showed that the community structure of NF-25% treatment was more different from other treatments, indicating that the fertilization method changed the community structure of soil bacteria. The results of a redundancy analysis showed that SOM, pH, AN, TN, and AP were the main factors affecting the microbial community structure. Overall, the reduction of nitrogen fertilizer with bio-organic fertilizer significantly increased the soil nutrient content, reduced the relative abundance and diversity of soil bacteria, increased the relative abundance of beneficial bacteria in the soil, changed the bacterial community structure of soil, increased production and created suitable soil conditions for the red raspberry growth.

## Introduction

Red raspberry (*Rubus idaeusl* L.) is an internationally popular third-generation fruit, which is rich in nutrients and contains anthocyanins, ellagic acid, salicylic acid, flavonoids and other active substances with anti-cancer [1], anti-aging [2], blood sugar [3] and lipid lowering effects [4, 5]. Nitrogen fertilization is an important element that affects the yield of red raspberry [6]. In recent years, farmers have blindly invested excessive nitrogen fertilizer in agricultural production in pursuit of high yield and profitability, which has led to a series of problems such as soil consolidation, soil nutrient loss, decreasing utilization rate of nitrogen fertilizer, and affecting soil bacterial diversity and community structure stability [7]. Therefore, moderate reduction of nitrogen fertilizer application is an inevitable trend for the future development of raspberry industry.

Soil bacteria are the most abundant and active group of microorganisms that can effectively promote the decomposition and release nutrients and organic matter. It also plays an important role in soil nitrogen morphological conversion [8, 9]. There are numerous microbial species in the soil, such as *Azospirillum*, *Streptomyces*, *Bacillus*, and *Pseudomonas*, which have been shown to have significant growth-promoting effects on plants, and soil-borne pathogens such as *Fusarium*, which can cause crop diseases [10]. The activity of soil bacteria is closely related to the physical and chemical properties of soil, and high application of nitrogen fertilizer would increase NO_3_^—^N content and decrease pH value in soil, which will affect the stability and diversity of soil bacterial community structure and change the supply capacity of effective soil nitrogen, ultimately affecting the growth and development of crops [11, 12]. Biofertilizers, as substances containing living microorganisms, have been shown to improve soil fertility and crop yields and can be used as an alternative to chemical fertilizers [13]. Many studies have reported that chemical fertilizer reduction with bio-organic fertilizer can improve soil nutrient content, increase the abundance and diversity of soil bacteria, and change the soil bacterial community structure, thus promoting crop yield [14–16]. In recent years, most studies have focused on medicinal value [17], deep processing product development [18], and improving plant resistance [19], while few studies have been reported on the effect of nitrogen fertilizer reduction with bio-organic fertilizer on bacterial community diversity and structure in red raspberry soil. But the effects of bio-organic fertilizer and nitrogen application on soil nutrients and their relation to microbial properties was uncertain.

In this study, we investigated whether different gradients of nitrogen fertilizer reduction with bio-organic fertilizer could affect the physical and chemical properties and bacterial composition of red raspberry garden soil. We expect to provide a theoretical basis for maintaining soil bacterial diversity and clarifying the reasonable amount of nitrogen fertilizer application in the field to create suitable soil conditions for red raspberry growth.

## Materials and methods

### Study site

The experiment was started in March 2021, and the test site was located in Sitai Village, Jingxiu District, Baoding City, Hebei Province, China (E115°41′, N38°84′), which is 22 m above sea level with tidal soils. The test site belongs to the warm temperate semi-humid semi-arid monsoon climate zone, dry and windy in spring and autumn, hot and rainy in summer. The average annual rainfall is 550mm. The annual average temperature is 12.3°C, and the highest and lowest temperatures are 41°C and -23°C, respectively. The soil organic matter (SOM), pH, total nitrogen (TN), alkali-hydrolyzable nitrogen (AN), available potassium (AK), and available phosphorus (AP) at a soil depth of 0–20 cm were 13.30 g kg^-1^, 7.88, 1.01 g kg^-1^, 9.05 mg kg^-1^, 106.88 mg kg^-1^, and 22.16 mg kg^-1^, respectively.

### Experimental design

Raspberries were planted in double-row strips in the trial site with 20 cm spacing between plants, 20 cm spacing between small rows and 2.5 m spacing between large rows. Double-season red raspberry "Polka" planted in 2019 was used as the test material. A total of six treatments with three replications of 3 m each (double rows), NF-100%: bio-organic fertilizer alone, NF-75%: conventional nitrogen application reduced by 75% + bio-organic fertilizer, NF-50%: conventional nitrogen application reduced by 50% + bio-organic fertilizer, NF-25%: conventional nitrogen application reduced by 25% + bio-organic fertilizer, CF: farmers’ conventional nitrogen application (without bio-organic fertilizer), CK: no nitrogen and bio-organic fertilizer applied. The fertilizer application rates for different treatments are shown in Table 1. Bio-organic fertilizer was used as the base fertilizer and was applied in the way of strip ditches on both sides of the planting row before sprouting, with a dosage of 30 t/hm^2^. Nitrogen fertilizer (urea N46%) was applied in equal amounts at 10 cm of initial stem growth and one week before flowering (total 225 kg/hm^2^). At 10 cm of primary stem growth, all treatments were fertilized with 120 kg/hm^2^ each of phosphorus (monocalcium phosphate P_2_O_5_ 12%) and potassium (potassium sulphate K_2_O 50%) fertilizer. Bio-organic fertilizer: fermented sheep manure as carrier, containing 120 million/g of multifunctional microorganisms (*Bacillus subtilis* 100 million/g, *Paenibacillus mucilaginosus* 0.1 billion/g). Nutrient status: pH: 8.93, SOM: 183.99 g/kg, total nitrogen (TN): 6.6 g/kg, total phosphorus (TP): 5.95 g/kg, total potassium (TK): 16.09 g/kg, alkali-hydrolyzable nitrogen (AN): 1.43 g/kg, also contains zinc, magnesium, boron and other trace elements and potassium xanthate, etc.

**Table 1:** Total amount of fertilizer applied to red raspberry under different fertilization treatments.

Treatment	Urea kg/hm^2^	Bio-organic fertilizer t/hm^2^
CK	0	0
NF-100%	0	30
NF-75%	56.25	30
NF-50%	112.5	30
NF-25%	168.75	30
CF	225	0

### Soil sampling and analysis

Destructive sampling was carried out on June 16, 2021, (bud stage) with three plots per treatment, and three red raspberry plants were randomly dug out whole from each plot, for a total of 54 plants. Soil samples from the same plot were evenly mixed and divided into two parts, one stored in a refrigerator at -80°C for high-throughput sequencing analysis, and one taken back to the laboratory for natural drying and sieved through 1 mm for soil chemical property determination. Soil organic matter (SOM), pH, total nitrogen (TN), alkali-hydrolyzable nitrogen (AN), available phosphorus (AP), and available potassium (AK) were measured as described by Institute of Soil Science, Chinese Academy of Sciences [20].

### Plant growth and yield

At the bud stage (June 16, 2021), three plants were selected from each plot for measurement, and 15 leaves from each plant were selected for leaf area measurement. The height and crown width of the red raspberry plants were measured with a tape measure and the ground diameter was measured with a vernier caliper. Leaf area was determined by scanning method, and the leaves were scanned using HP Scanjet 2400 scanner. The evaluation parameter was area yield (kg/hm^2^), carried out by weighing of the total fruits produced by five plants of each treatment along the entire harvesting period (September 28, 2021).

### DNA extraction and 16S rRNA gene amplicon sequencing

Total genomic DNA samples were extracted using the OMEGA Soil DNA Kit (M5635 02) (Omega Bio Tek, Norcross, GA, USA), following the manufacturer’s instructions, and stored at -20°C prior to further analysis. The quantity and quality of extracted DNAs were measured using a NanoDrop NC2000 spectrophotometer (Thermo Fisher Scientific, Waltham, MA, USA) and agarose gel electrophoresis, respectively.

PCR amplification of the bacterial 16S rRNA genes V3–V4 region was performed using the forward primer 338F (5’-ACTCCTACGGGAGGCAGCA-3’) and the reverse primer 806R (5’-GGACTACHVGGGTWTCTAAT-3’). Sample-specific 7-bp barcodes were incorporated into the primers for multiplex sequencing. The PCR components contained 5 μl of buffer (5×), 0.25 μl of Fast pfu DNA Polymerase (5 U/μl), 2 μl (2.5 mM) of dNTPs, 1 μl (10 uM) of each Forward and Reverse primer, 1 μl of DNA Template, and 14.75 μl of ddH_2_O. Thermal cycling consisted of initial denaturation at 98°C for 5 min, followed by 25 cycles consisting of denaturation at 98°C for 30 s, annealing at 53°C for 30 s, and extension at 72°C for 45 s, with a final extension of 5 min at 72°C. PCR amplicons were purified with Vazyme VAHTSTM DNA Clean Beads (Vazyme, Nanjing, China) and quantified using the Quant-iT PicoGreen dsDNA Assay Kit (Invitrogen, Carlsbad, CA, USA). After the individual quantification step, amplicons were pooled in equal amounts, and pair-end 2×250 bp sequencing was performed using the Illlumina NovaSeq platform with NovaSeq 6000 SP Reagent Kit (500 cycles) at Shanghai Personal Biotechnology Co., Ltd (Shanghai, China).

Microbiome bioinformatics were performed with QIIME2 2019.4 with slight modification according to the official tutorials (https://docs.qiime2.org/2019.4/tutorials/tutorials/). Briefly, raw sequence data were demultiplexed using the demux plugin following by primers cutting with cutadapt plugin [21]. Sequences were then quality filtered, denoised, merged and chimera removed using the DADA2 plugin [22]. Non singleton amplicon sequence variants (ASVs) were aligned with mafft and used to construct a phylogeny with fasttree2. Alpha diversity metrics Chao1, Observed species, Shannon, Simpson, Faith’s PD, Pielou’s evenness and Good’s coverage, beta dive rsity metrics weighted UniFrac, unweighted UniFrac, Jaccard distance, and Bray Curtis dissimilarity were estimated using the diversity plugin with samples. Taxonomy was assigned to ASVs using the classify sklearn na naïve Bayes taxonomy classifier in feature classifier plugin against the SILVA Release 132 Database.

### Data analysis and processing

Excel 2010 software was used for data processing and graphing. SPSS 26.0 was used for data analysis, and One-way analysis of variance was performed using ANOVA (p < 0.05). Sequence data analyses were mainly performed using QIIME2 and R packages (v3.2.0). ASV-level alpha diversity indices, such as Chao1 richness estimator, Observed species, Shannon diversity index, Simpson index, Faith’s PD, Pielou’s evenness and Good’s coverage were calculated using the ASV table in QIIME2, and visualized as box plots. ASV-level ranked abundance curves were generated to compare the richness and evenness of ASVs among samples. Beta diversity analysis was performed to investigate the structural variation of microbial communities across samples using UniFrac distance metrics [23, 24] and visualized via principal coordinate analysis (PCoA). To further compare the differences in species composition between samples, the clustering results for each sample as well as for each taxonomic unit were calculated using the R script, and the abundance data for the top 50 genera in terms of mean abundance were used to create heat maps. LEfSe (Linear discriminant analysis effect size) was performed to detect differentially abundant taxa across groups using the default parameters [25]. The Redundancy analysis (RDA) and heat maps were performed by the genescloud tools, a free online platform for data analysis (https://www.genescloud.cn).

## Results

### Soil physical and chemical properties

As shown in Table 2, nitrogen fertilizer reduction with bio-organic fertilizer had significant effects on soil chemical properties. the NF-25%, NF-50%, and NF-75% treatments significantly increased SOM, TN, AN, AP, and AK and decreased soil pH compared to CK. The content of SOM, TN, AN, AP and AK were much higher in the NF-25% treatment than in the other treatments. The results showed that nitrogen fertilizer reduction with bio-organic fertilizer could significantly improve soil fertility, and the best overall soil fertility effect was found in NF-25% treatment.

**Table 2:** Effects of nitrogen reduction combined with bio-organic fertilizer on soil chemical properties.

Treatments	pH	SOM g/kg	TN g/kg	AN mg/kg	AP mg/kg	AK mg/kg
CK	7.95±0.03ab	15.13±0.69b	1.27±0.06c	9.98±1.06c	20.90±0.78c	114.95±10.92c
NF-100%	7.99±0.05a	15.82±1.38b	1.46±0.11bc	11.61±1.61bc	22.28±5.35bc	135.91±11.33bc
NF-75%	7.82±0.06bc	19.49±2.78ab	1.72±0.13ab	13.93±2.09bc	33.09±7.20ab	154.07±10.06ab
NF-50%	7.79±0.14c	18.11±3.79ab	1.79±0.22a	15.53±2.79b	32.40±9.41abc	167.62±15.19a
NF-25%	7.73±0.08c	22.70±1.82a	1.94±0.13a	20.90±1.39a	38.63±5.92a	169.85±18.60a
CF	7.99±0.08a	15.59±2.21b	1.75±0.17a	14.16±2.45bc	22.68±4.27bc	126.32±8.76c

The data are the mean ± standard deviation (n = 3); different lowercase letters after the values in the same column indicate significant differences (P<0.05) The same letter represents no significant difference. SOM: soil organic matter, TN: total nitrogen, AN: alkali-hydrolyzable nitrogen, AP: available phosphorus, AK: available potassium.

### Comparison of the community composition at the phylum level

**Fig 1:**
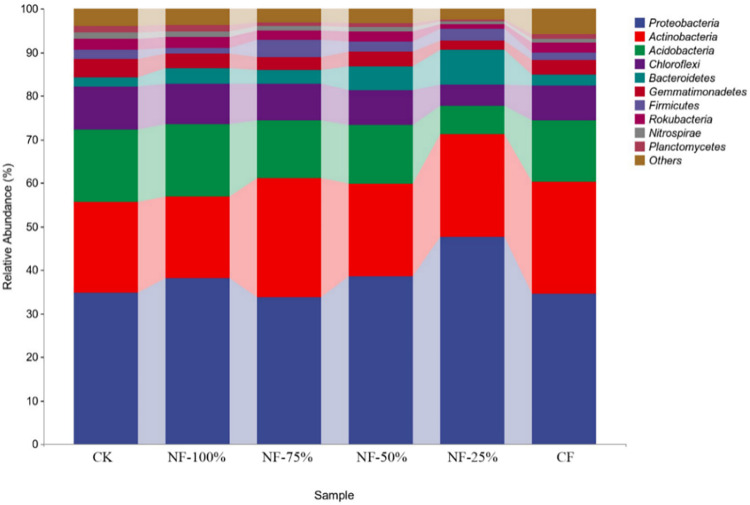
Taxonomic composition of phylum of different treatments.

As shown in Fig 1, a total of 40 taxa were obtained at the phylum level, and the dominant phylum in all samples were *Proteobacteria* (33.68%-47.67%), *Actinobacteria* (18.81%-27.40%), *Acidobacteria* (6.70%-16.66%), *Chloroflexi* (4.67%-9.86%), *Bacteroidetes* (2.26%-4.67%), which accounted for more than 85% of the relative abundance of the bacterial community. Among these dominant clades, *Proteobacteria* were the most abundant in the NF-25% (46.67%) treatment, but less abundant in the CK (34.87%) and CF (34.48%) treatments. On the contrary, *Acidobacteria* and *Chloroflexi* were higher in CK (16.54%, 9.86%) and NF-100% (16.66%, 9.14%) treatments and in NF-25% (6.70%, 4.67%) treatment the lowest. *Actinobacteria*, the second most abundant phylum, had the highest content in the NF-75% (27.40%) treatment. Bacteroidetes were most abundant in the NF-25% (8.13%) treatment, but the lowest in the CK (2.26%) and CF (2.40%) treatments.

### Soil bacterial diversity

**Fig 2:**
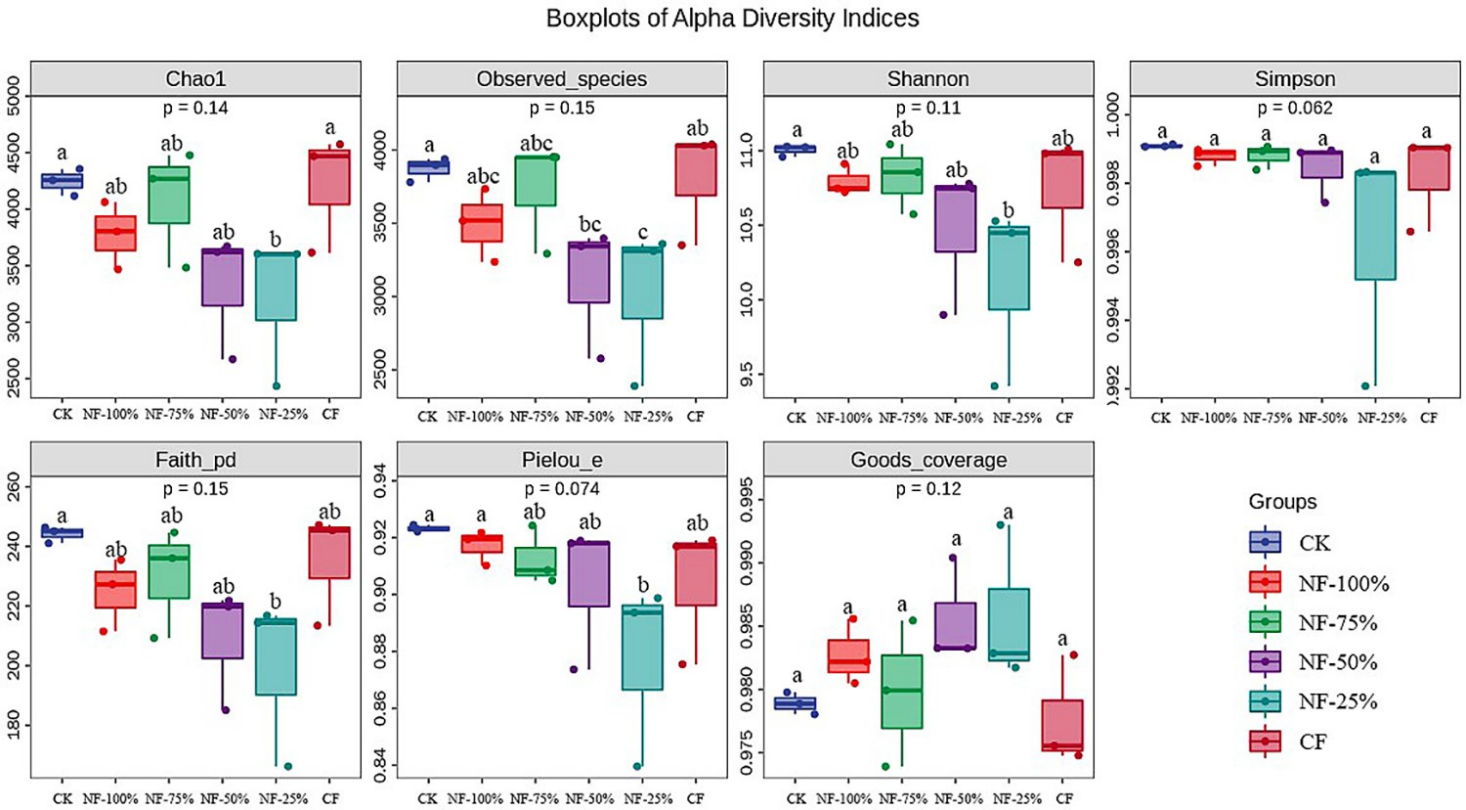
Alpha diversity index of soil bacterial community under different treatments.

Alpha diversity of the soil bacterial community under different fertilization treatments is shown in Fig 2. The Good’s coverage index of all treatments was greater than 0.97, which also indicates that the sequencing ability can reflect the bacterial community characteristics of soil samples more accurately. CK of Chao1, Observed species index was the highest, followed by CF. Meanwhile, the Shannon and Simpson indices of CK treatment were higher than other treatments. Not coincidentally, the highest bacterial Pielou’s evenness and Faith’s PD indices were found in CK soil samples. It may be that bio-organic fertilizer contains a large number of beneficial active bacteria and organic matter, which can cause nutrient robbery among microorganisms, and the dominant population inhibits the reproduction of other populations.

### Differential analysis of bacterial communities

**Fig 3:**
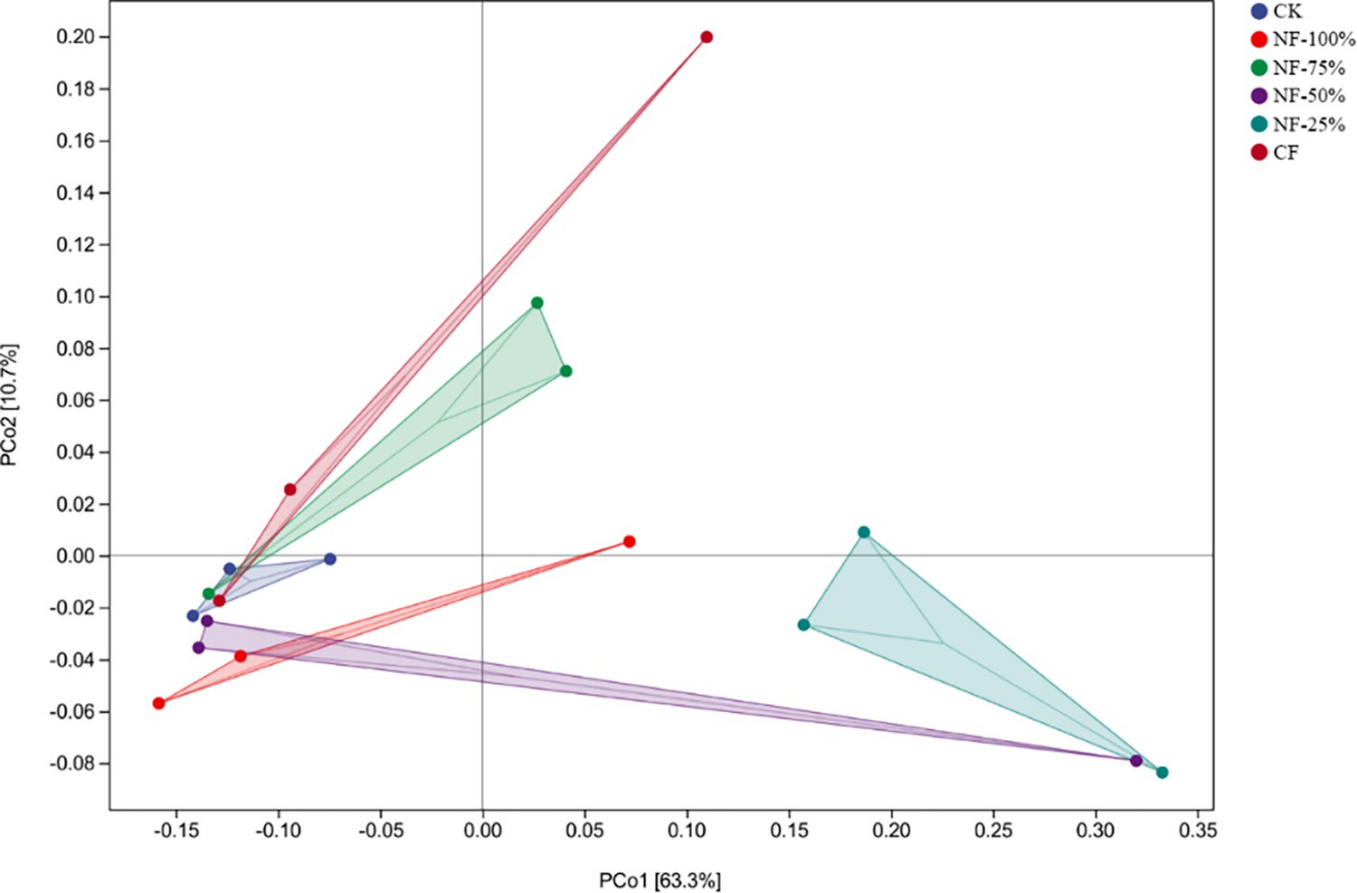
PCoA analysis of soil bacterial community under different treatments.

PCoA analysis based on weighted-unifrac distance algorithm for soil samples with different fertilization treatments. As shown in Fig 3, the first principal component (PCo1) explains 63.3% of the variation in the bacterial community and the second principal component (PCo2) explains 10.7% of the variation. The closer the projection distance of the samples on the axes, the more similar the community composition of these two samples in the corresponding dimension. Similarly, the differences in community composition among CK, NF-50% and NF-100% were smaller. NF-25% was far from the other treatments, indicating that its bacterial community composition was more different from the other treatments.

**Fig 4:**
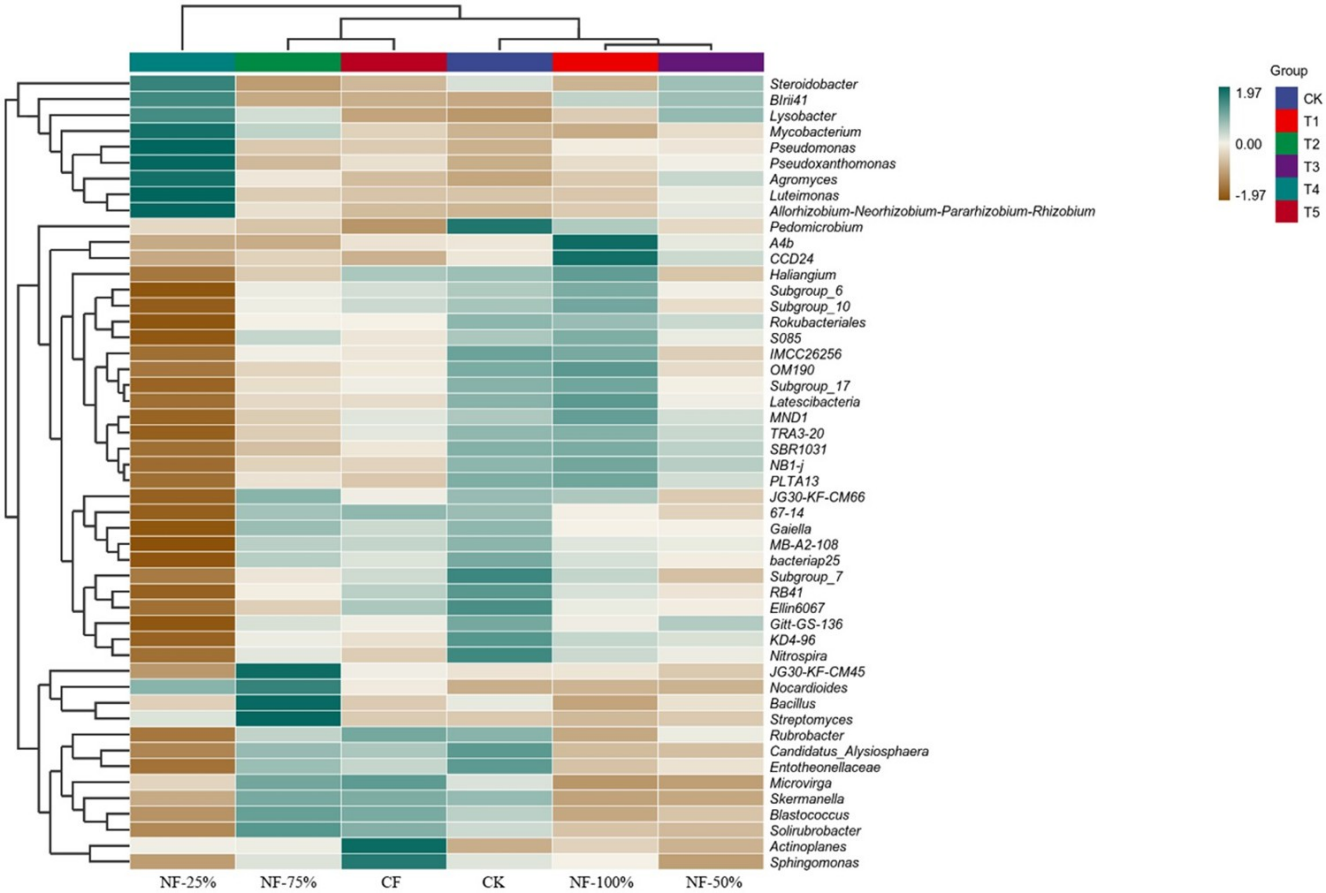
Cluster analysis heat map of bacterial community structure of different treatments at genus level.

To further explore the effect of nitrogen fertilizer reduction with bio-organic fertilizer on the soil community structure of red raspberry, cluster analysis was performed on the dominant soil bacteria at the genus level (Fig 4). CK, NF-100% and NF-50% were clustered into the same group, while NF-75% and CF were clustered into another group. The dominant genera of CK, NF-100% and NF-50% were *Pedomicrobium*, *A4b* and *CCD24*. In the other group, the dominant genera for NF-75% and CF were *Bacillus*, *Streptomyces*, *JG30-KF-CM45*, *Sphingomonas* and *Actinoplanes*. The dominant genera for NF-25% treatment was *Allorhizobium-Neorhizobium-Parararhizobium-Rhizobium*, *Pseudoxanthomonas*, *Mycobacterium*, *Lysobacter*, *Luteimonas*, *Agromyces*, and *Pseudomonas*, which differed significantly from the dominant genera in the other treatments.

**Fig 5:**
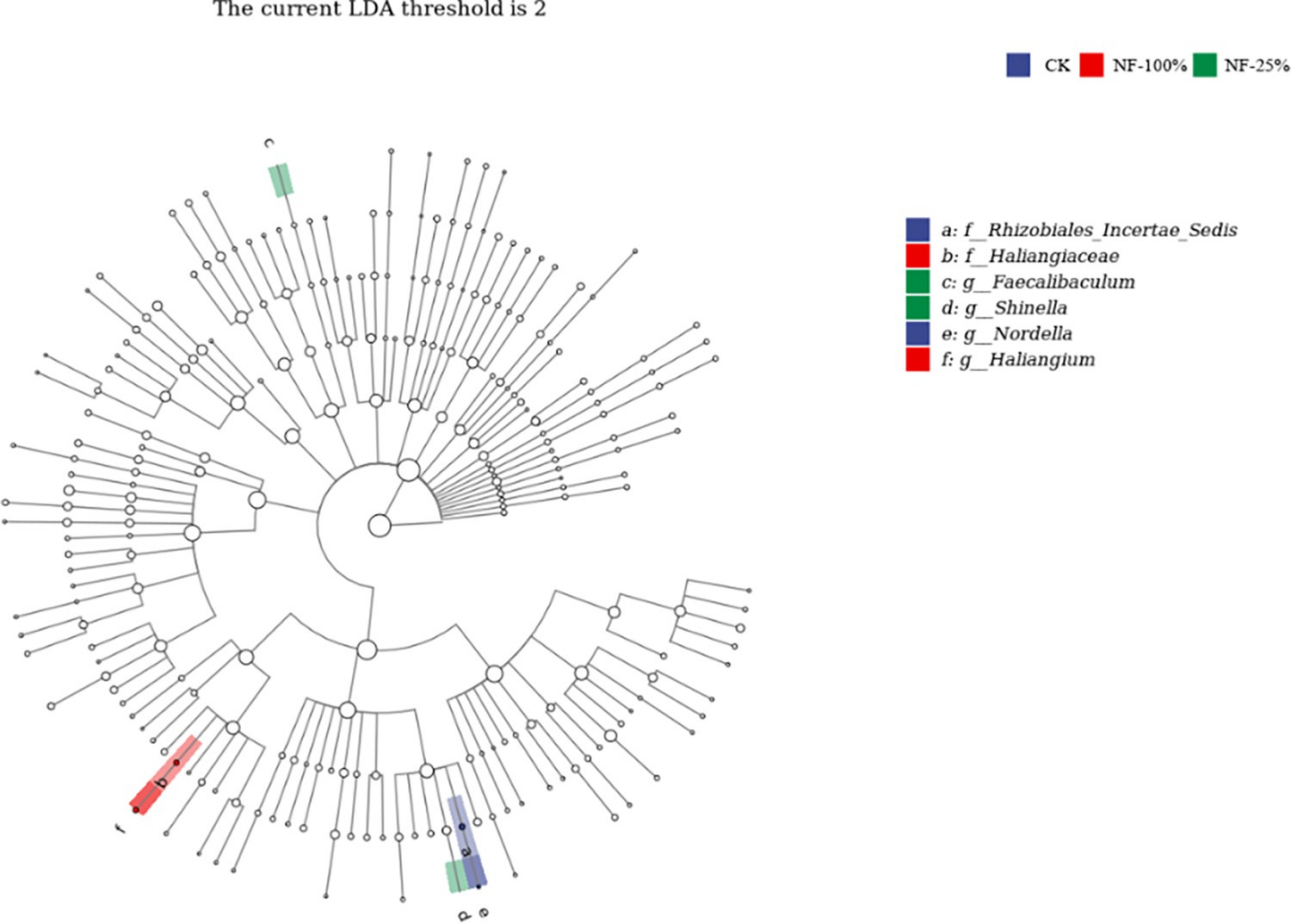
Phylogenetic tree of soil bacterial community under different fertilization treatments.

LefSe linear discriminant analysis (LDA) was used to detect populations with significant differences in abundance between fertilization treatments (Fig 5). A total of six bacterial evolutionary branches showed significant differences in the different soil samples with an LDA threshold of 2. In CK, the relative abundance of *Rhizobiales_Incertae_Sedis*, *Nordella* was significantly higher than the other treatments. The relative abundance of *Haliangiaceae*, *Haliangium* was higher in the NF-100% treatment compared to the other treatments. NF-25% treatment of *Faecalibaculum*, *Shinella* was more enriched than other treatments.

### Relationship between bacterial community structure and environmental factors

**Fig 6:**
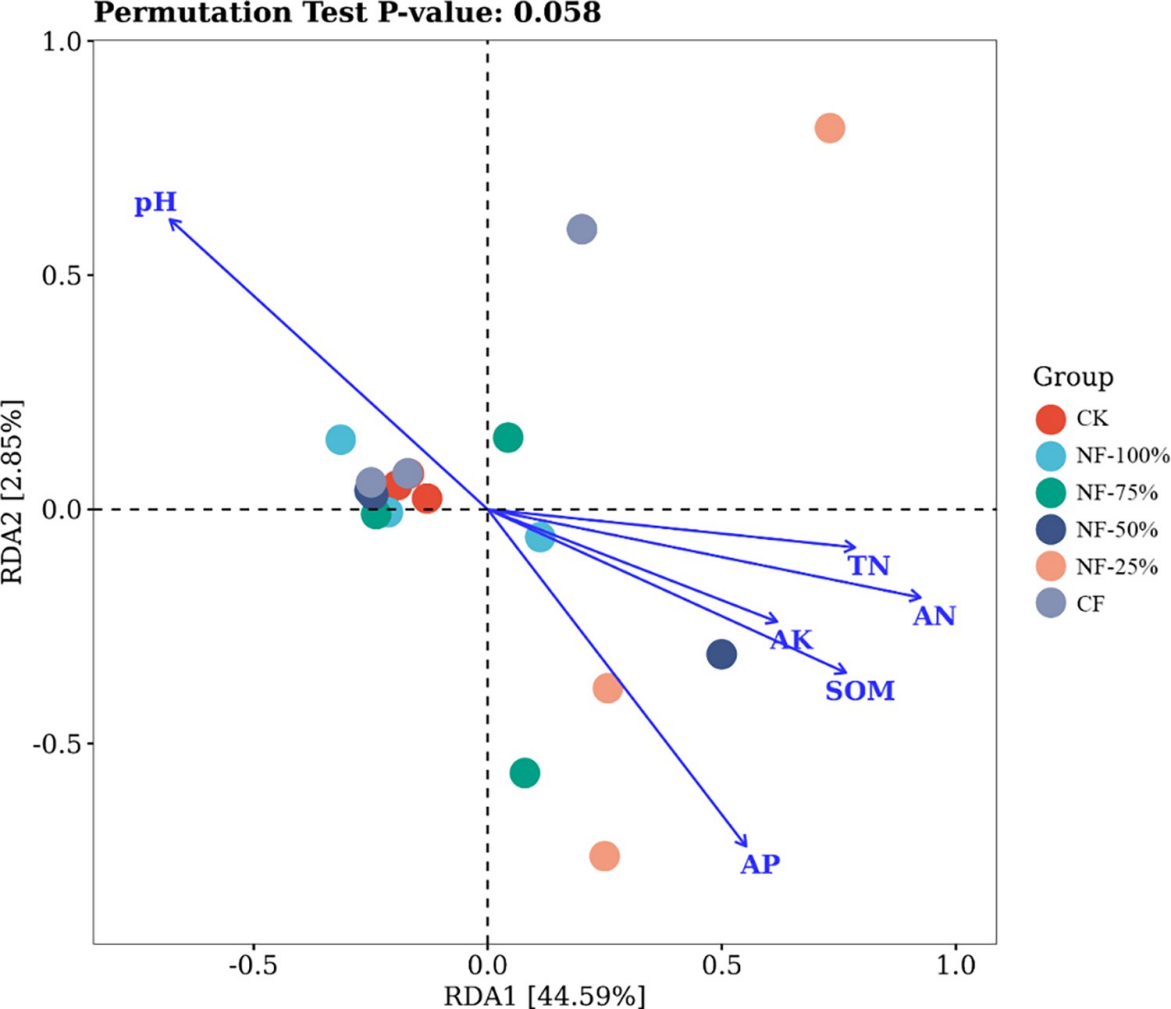
RDA analysis of soil bacterial community and soil chemical *properties* under different fertilization treatments at genus level.

To investigate the effect of soil chemical properties on the bacterial community of red raspberry, RDA analysis was performed on the soil bacterial community and soil chemical properties of different fertilization treatments at the genus level. As shown in Fig 6, the two RDA components together explained 47.44% of the variation in bacterial community structure, with the first component (RDA1) explaining 7.70% of the variation; the second component (RDA2) explained 6.02% of the variation. As shown in Table 3, soil chemical properties had significant effects on soil bacterial community structure, with pH (P = 0.003), AN (P = 0.003), AP (P = 0.004) and SOM (P = 0.009) having highly significant effects (P < 0.01) on the bacterial community composition in the soil, and TN (P = 0.013) had a significant effect (P < 0.05) on the composition of the soil bacterial community.

**Table 3:** P value of correlation between genus level bacterial community and soil chemical properties.

Soil chemistry properties	RDA1	RDA2	*r*	*p*
pH	-0.8446	0.5353	0.5208	0.003
TN	0.9925	-0.1221	0.4275	0.013
AN	0.9826	-0.1855	0.6123	0.003
AP	0.7558	-0.6546	0.4740	0.004
AK	0.9557	-0.2942	0.2978	0.072
SOM	0.9435	-0.3313	0.4750	0.009

**Fig 7:**
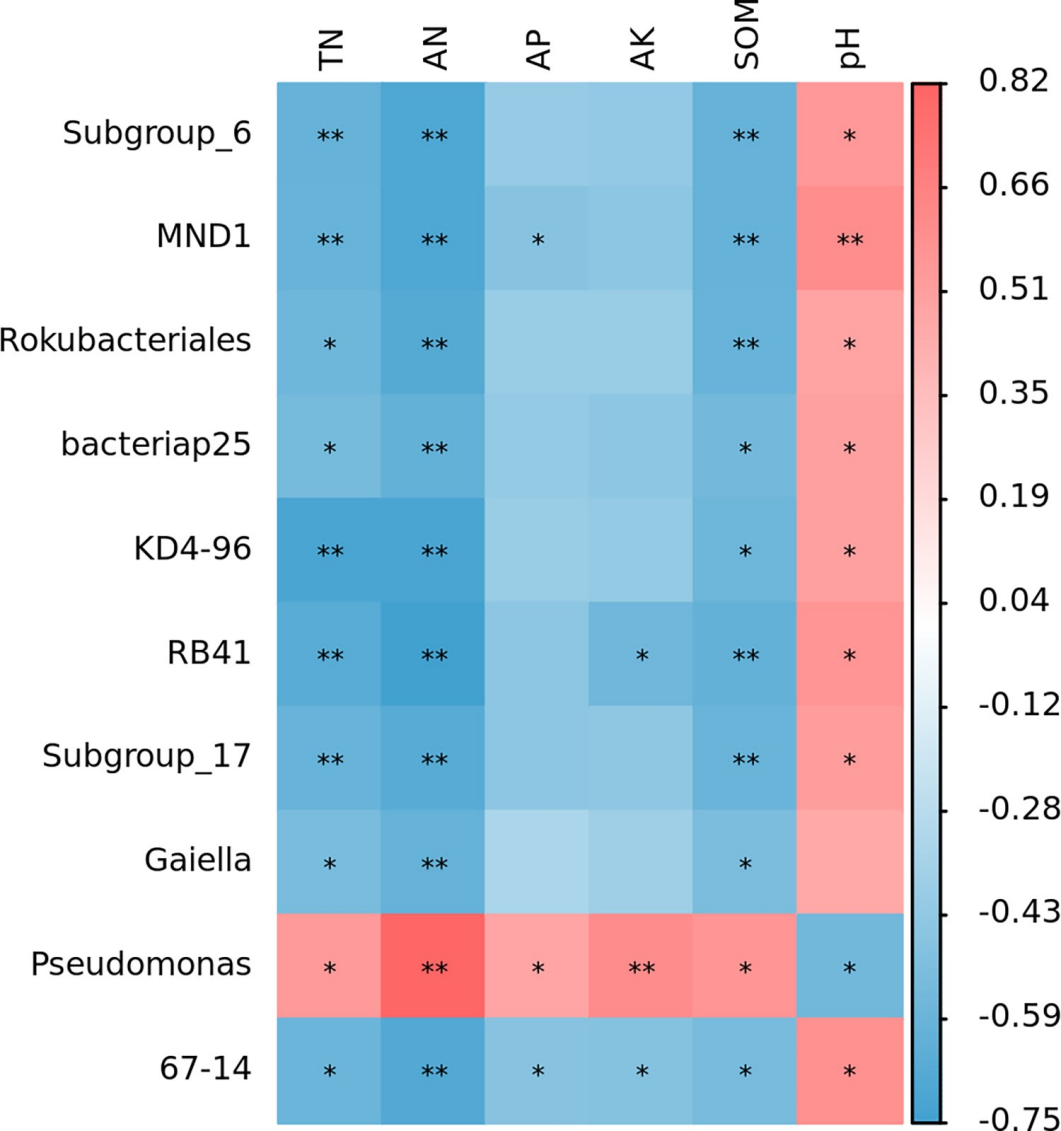
Heat map of the association between major soil bacterial taxa and soil chemical properties at the genus level. * indicates significant correlation P<0.05; ** indicates highly significant correlation P<0.01.

Correlation analysis of major bacterial taxa and soil chemical properties in the soil of red raspberry garden, as shown in Fig 7. Subgroup_6、MND1、Rokubacteriales、bacteriap25、KD4−96、RB41、Subgroup_17、Gaiella、67−14 ***showed*** significant negative correlations (P < 0.05) with TN and SOM, highly significant negative correlations (P < 0.01) with AN, but significant positive correlated with pH (P < 0.05). Pseudomonas was significantly and positively correlated with TN, AP and SOM (P < 0.05), and highly significantly positively correlated with AN and AK (P < 0.01), but significantly negatively correlated with pH (P < 0.05).

### Red raspberry plant growth and yield

As shown in Table 4, nitrogen fertilizer reduction with bio-organic fertilizer increased red raspberry plant height, ground diameter, crown size, and leaf area. Among them, NF-25% treatment had the most significant effect on promoting the growth of red raspberry trees.

**Table 4:** Effect of nitrogen fertilizer reduction with bio-organic fertilizer on the growth of red raspberry plants.

Treatments	Plant height	Ground path	Crown	Leaf area
	cm	mm	cm	cm^2^
CK	61.33±8.50a	9.07±2.37b	29.67±0.58a	117.56±12.68c
NF-100%	97.00±6.08a	13.27±1.57a	34.28±3.21a	181.65±9.78ab
NF-75%	90.00±6.24a	14.32±1.69a	33.31±3.24a	200.46±9.24ab
NF-50%	84.66±15.53a	13.21±2.01a	34.53±3.79a	205.83±25.54a
NF-25%	103.00±16.64a	15.85±3.72a	34.67±3.21a	212.91±11.62a
CF	88.33±3.21a	13.46±0.40a	33.00±1.00a	173.38±22.12b

**Fig 8:**
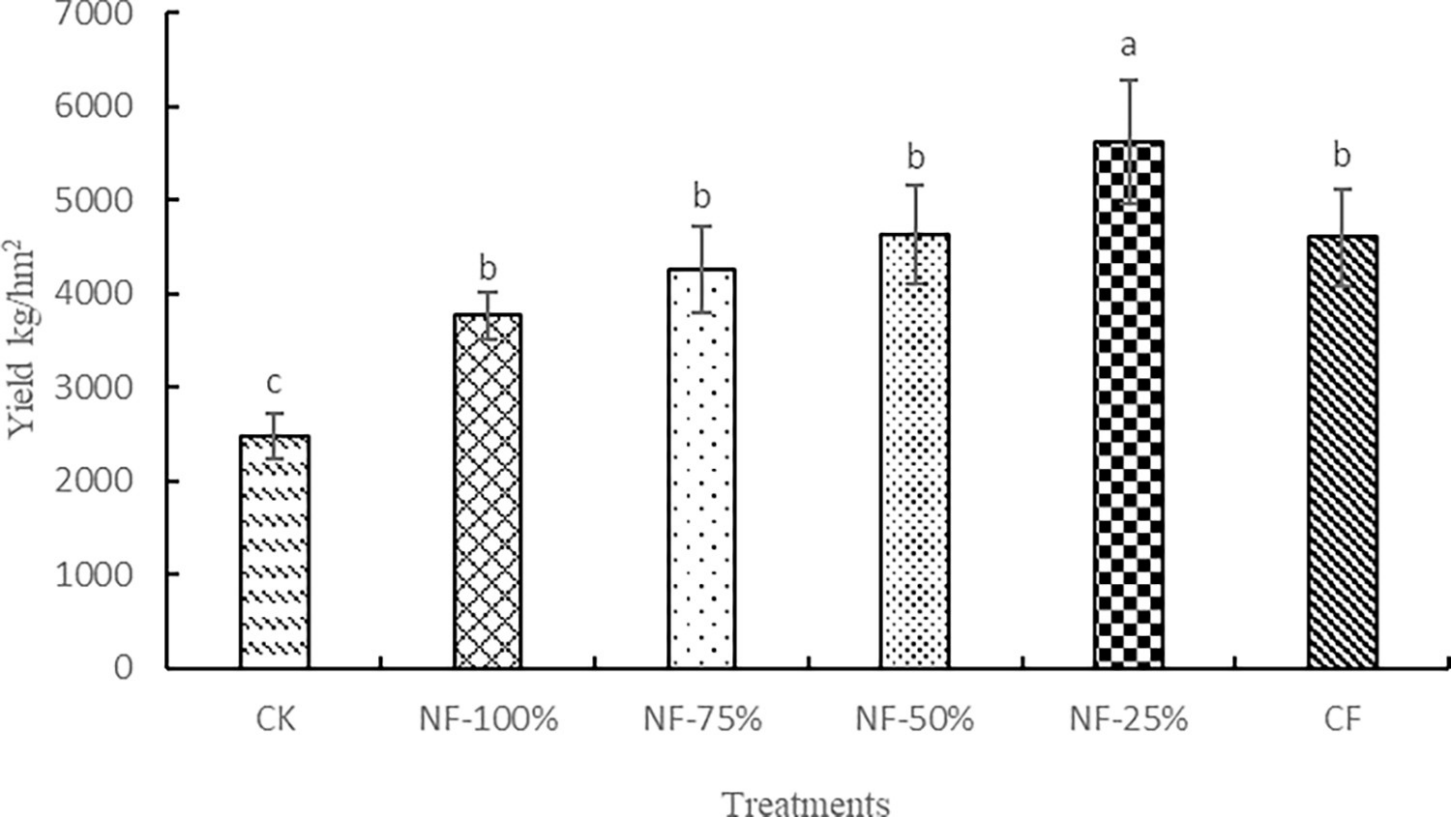
Effect of nitrogen reduction combined with microbial organic fertilizer on fruit yield of red raspberry.

The effects of different fertilizer treatments on red raspberry yield are shown in Fig 8. Both NF-50% and NF-25% treatments increased the yield of red raspberry compared to CF treatment. The yield of red raspberry in NF-25% treatment was significantly higher than the other treatments, 5621.20 kg/hm^2^, which was 22.15% higher than CF. It showed that the reduction of nitrogen fertilizer with microbial organic fertilizer could significantly increase the yield of red raspberry. The lower overall yield of red raspberry may be due to the effect of long-term rainfall in the test site during the fruiting period, soil nutrient loss, and reduced photosynthetic efficiency of red raspberry, resulting in insufficient nutrient supply.

## Discussion

In recent years, excessive application of nitrogen fertilizer in agricultural production has been widespread, leading to adverse effects such as crop yield reduction, decreased soil fertility, reduced soil enzyme activity, and reduced microbial community diversity [26, 27]. Nitrogen fertilizer reduction with organic fertilizer not only increases yield, improves quality, and enriches soil but also improves soil quality, soil microbial diversity, and community structure [28, 29]. In this study, nitrogen fertilizer reduction with bio-organic fertilizer treatment lowered soil pH and increased the content of soil organic matter, total nitrogen, alkali-hydrolyzable nitrogen, available phosphorus and available potassium contents, in which NF-25% treatment had the highest nutrient content. These results are consistent with the studies of Zhang [30] and He [31]. The reason is that the plant growth promoting rhinoacteria (PGPR) in bio-organic fertilizers have physiological functions such as phosphorus solubilization, potassium solubilization and nitrogen fixation, which release soluble potassium and phosphorus for crop uptake and utilization through certain pathways. It can also supply nitrogen by converting N_2_ to NH_3_ [32, 33]. Application of bio-organic fertilizers enriches the soil with specific microorganisms, attracts beneficial bacteria, and promotes their reproduction, thus increasing soil nutrients.

In this study, the soil bacteria in the red raspberry garden mainly consisted of *Proteobacteria*, *Actinobacteria*, *Acidobacteria*, *Chloroflexi* and *Bacteroidetes*, which was consistent with the findings of Chen [34], Sun [35] and Zhang [36]. *Proteobacteria* plays an important role in the soil nitrogen cycle and helps to improve soil fertility and promote plant carbon and nitrogen uptake [37, 38]. *Acidobacteria* has the role of promoting soil carbon cycle [39]. Campbell [40] et al. found a positive correlation between *Acidobacteria* and soil pH. In this study, the abundance of Proteobacteria increased significantly under nitrogen fertilizer reduction treatment, but the abundance of *Acidobacteria* decreased significantly. The reason was that nitrogen addition induces an increase in the relative abundance of soil *Proteobacteria* and a decrease in the relative abundance of *Acidobacteria* thus changing the community structure of soil bacteria, which were consistent with those of previous studies [11, 41]. *Actinobacteria* have the ability to degrade mineral and organic matter and play a key role in promoting plant growth [42]. The differences in the relative abundance of *Actinobacteria* among fertilization treatments in this study were small. Studies have shown *Proteobacteria* and *Bacteroidetes* to be copiotrophic bacteria, *Acidobacteria* and *Chloroflexi* to be oligotrophic bacteria [43, 44]. In this study, the relative abundance of *Chloroflexi* in NF-25% treatment was the lowest, and most of *Chloroflexi* were anaerobic bacteria, which means that the reduced abundance increased soil permeability and accelerated the rate of soil nutrient mineralization, thus facilitating nutrient uptake and utilization by the root system [45, 46].

Soil bacterial diversity and richness were reduced in this study in the treatment of nitrogen fertilizer reduction with bio-organic fertilizer, which was inconsistent with the results of Shang [47]. and consistent with the results of Cai [48]. The main reason may be the additional application of bio-organic fertilizer on top of the nitrogen fertilizer reduction treatment, which contains a large amount of beneficial active bacteria and organic matter. It could cause nutrient robbery among microorganisms and the dominant population inhibited the reproduction of other populations [49]. The application of organic fertilizers introduces exogenous microorganisms that disrupt the dynamic balance of the original soil microorganisms and cause changes in the entire soil microbiota [50].

Differences in environmental factors can affect plant growth and development and the structure of their soil microbial communities [51, 52]. Based on the results of this experiment, PCoA plots clearly distinguished the differences in soil bacterial microbial communities among fertilization treatments. These results were consistent with the findings of most long-term fertilization trials [53], in which application of different levels of N fertilizer with bio-organic fertilizer affected the soil microbial community structure to different degrees. In this study, LEfSe analysis and cluster analysis of the soil bacterial community revealed that the dominant genera differed significantly among treatments. CK and NF-100% were clustered into the same group, while NF-75%, CF and NF-50% were clustered into another group. However, the NF-25% treatment was significantly different from the dominant genus in the other treatments. The dominant genus of CK, *Pedomicrobium*, has the ability to retain iron and manganese in the soil environment, preventing the harmful effects of nutrient loss to plants [54]. *Sphingomonas* [55], a dominant genus in CF treatment, has a unique ability to degrade polycyclic aromatic hydrocarbons (PAHs), often present in petroleum-contaminated soils. In addition, studies have shown that *Sphingomonas* promote the growth of plants in saline areas [56]. It is possible that the soil of the test site is alkaline, and in the absence of nitrogen fertilization and organic fertilization, the red raspberry stimulated *Sphingomonas* to be in a dominant position in the soil in response to the unfavorable external conditions. *Bacillus* and *Streptomyces* are the dominant genera enriched in the NF-75% treatment. *Bacillus* has biocontrol function, while *Bacillus* and *Streptomyces* can promote the formation and stabilization of soil aggregates, reduce the evaporation of water from the soil surface, contribute to the reduction of soil salinity, and improve the environmental characteristics in the soil [57]. The dominant genus in the NF-25% treatment, Pseud*omonas*, has the ability to decomposing proteins, fats, and soil humus and which is a bacterium that promotes plant growth [58, 59]. *Luteimonas* and *Lysobacter* are mercury-tolerant bacteria in soil and are resistant to mercury contamination [60]. Members of *Shinella* can effectively degrade micro-contaminants including polycyclic aromatic hydrocarbons (PAHs), among other micropollutants [61]. Anti-pollutant bacteria enrichment may be related to the application of nitrogen fertilizer, which increases the diversity of heavy metal-tolerant and nitrogen-fixing bacteria such as Pseudomonas, Azospirillum [62]. Studies have shown that nitrogen-fixing bacteria was tolerant to heavy metals and could remediate contaminated soil [63]. The addition of nitrogen fertilizer to external sources of petroleum polluted environment could promote the degradation of petroleum hydrocarbons. However, excessive application of nitrogen fertilizer not only caused an increase in nitrate content in the environment, resulting in secondary pollution, but also increased remediation costs [64, 65]. Theoretically, if a significant proportion of the bacterial population was tolerant to a high concentration of metal contaminant, then the judgment was made that the soil was negatively affected by the presence of the metal. However, it had also been found that the number of heterotrophic nitrogen-fixing bacteria was significantly reduced in contaminated soil. Therefore, the reasons for the enrichment of such bacteria need to be further studied. The results of this study showed that nitrogen fertilizer reduction with bio-organic fertilizer could promote the decomposition of organic matter and soil pollutants and changed the community structure of soil bacteria.

Changes in soil chemistry under the influence of nitrogen fertilizer reduction with bio-organic fertilizer had different contributions to microbial taxa. In this study, key factors affecting the structure and composition of soil bacterial communities were revealed for pH, organic matter, total nitrogen, alkali-hydrolyzable nitrogen, and available phosphorus content. This was in general agreement with the results of Yang [66] and Zhou [67], who showed that soil pH, organic carbon and total nitrogen content significantly affected the community structure of bacteria. This indicates that the treatment of nitrogen fertilizer reduction with bio-organic fertilizer not only increased the nutrient content in the soil, but also affected the community composition of bacteria.

Microbial organic fertilizer affects the bacterial structure and abundance of the soil. The addition of microbial organic fertilizer provides sufficient substrate for soil microorganisms, which facilitates microbial colonization at the rhizosphere, leading to an increase in rhizosphere microbial diversity [68]. The large number of microorganisms carried by the exogenously added bioorganic fertilizer itself was also an important reason for the increased bacterial community diversity under the N fertilizer reduction treatment [69, 70]. However, Li [71] found that the bacterial species carried by organic manure itself was significantly different from the composition of soil bacterial community after the application of chemical fertilizer combined with organic manure. Only a small percentage of the bacterial species in the soil samples came from the organic manure itself. Shi [72] found that Shannon diversity decreased, and bacterial community composition shifted in the bioorganic fertilizer treatment compared to non-fertilizer treatment. There was no significant difference in species richness between the two treatments. The addition of a bioorganic fertilizer also altered the metabolomic profile of rhizosphere soils and decreased the composition of many compounds belonging to the classes of phenolic acids, lipids, and flavonoids. Soil microorganisms mainly control most of the reactions in the nitrogen cycle [73]; Therefore, strong interactions between bacterial flora may occur after the addition of bioorganic fertilizers. A decrease in bacterial diversity might be due to plant response to changes in soil environmental conditions caused by bioorganic fertilization leading to the enrichment of a specific subset of functional microbiota in the rhizosphere [72]. At present, there were many researches [74–76] on the regulation of bioorganic fertilizer on soil bacterial diversity and structure, but there was lack of researches on the decrease of soil bacterial diversity caused by the application of bioorganic fertilizer. Therefore, this part will be the direction of our future research.

## Conclusion

NF-25% treatment not only significantly increased the yield of red raspberry and the contents of soil organic matter, total nitrogen, alkali-hydrolyzable nitrogen, available phosphorus and available potassium, and reduced soil pH, but also increased the relative abundance of soil beneficial bacteria and changed the community structure of soil bacteria, and created soil environmental conditions suitable for the growth of red raspberry. However, the application of bio-organic fertilizer reduces soil bacterial richness and diversity, so we still need to conduct more research in the future to explore the combined benefits of bio-organic fertilizer application.

## References

[1] Zhang HP, Li GD, Wei JF, Wang YF, Dang SW, Fang X, et al. Effect of red raspberry extract on proliferation of human hepatocellular carcinoma SMMC-7721 cells. China Cancer. 2019; 28(02): 155–160. Chinese.

[2] Mansour M, Salah M, Xu X. Effect of microencapsulation using soy protein isolate and gum arabic as wall material on red raspberry anthocyanin stability, characterization, and simulated gastrointestinal conditions. Ultrasonics Sonochemistry. 2020; 63(C): 104927. doi: 10.1016/j.ultsonch.2019.104927 31952001

[3] He K, Song S, Zou Z, Feng M, Wang D, Wang Y, et al. The Hypoglycemic and Synergistic Effect of Loganin, Morroniside, and Ursolic Acid Isolated from the Fruits of Cornus officinalis. Phytotherapy Research Ptr. 2016; 30(2): 283–91. doi: 10.1002/ptr.5529 26619955

[4] Jin Z, Kuang H, Wang J. Comprasion of the hypolipidemic activities of water extractions and ethanol extractions from red resberry. Science and Technology of Food Industry. 2017; 38(20): 307–311. Chinese.

[5] Jing X, Zhang N, Zhao L, Zhou J, Wu W, Zhang W, et al. Effect of soaked and fermented raspberry wines on the liver in mice. Food Bioscience; 2022, 47.

[6] Rizzi R, Silvestre WP, Rota LD, Pauletti JF. Raspberry production with different NPK dosages in South Brazil. Scientia Horticulturae. 2020; 261(C): 108984–108984.

[7] Xiang F, Li W, Liu HY, Ying X, Zeng ZX, Zhou LY. Influence of nitrogen fertilizer reduction on the structure of bacterial community in tea garden soil. Biotechnology Bulletin. 2021; 37(06): 49–57. Chinese.

[8] Delgado–Baquerizo M, Grinyer J, Reich P B, Singh B K. Relative importance of soil properties and microbial community for soil functionality: insights from a microbial swap experiment. Functional Ecology, 2016; 30(11): 1862–1873.

[9] Barbi F, Prudent E, Vallon L, Buée M, Dubost A, Legout A, et al. Tree species select diverse soil fungal communities expressing different sets of lignocellulolytic enzyme-encoding genes. Soil Biology and Biochemistry. 2016; 100: 149–159.

[10] Yang Z, Dai C, Wang X, Li X. Advance in research on rhizosphere microbial mechanisms of crop soil-borne fungal diseases. Acta Pedologica Sinica, 2019; 56(01): 12–22. Chinese.

[11] Li Z, Luo C, Qiu H, Fu X, Deng D, Zhang C, et al. Effects of continuous nitrogen application on bacterial community structure and denitrification in the rhizosphere of potato. Acta Prataculturae Sinica. 2020; 29(06): 105–116. Chinese.

[12] Fierer N, Ladau J, Clemente JC, Leff J W, Owens SM, Pollard KS, et al. Reconstructing the Microbial Diversity and Function of Pre-Agricultural Tallgrass Prairie Soils in the United States. Science. 2013; 342(6158): 621–624. doi: 10.1126/science.1243768 24179225

[13] Wang J, Liu L, Gao X, Hao J, Wang M. Elucidating the effect of biofertilizers on bacterial diversity in maize rhizosphere soil. PloS one. 2021; 16(4): e0249834–e0249834. doi: 10.1371/journal.pone.0249834 33891590 PMC8064744

[14] Liu L, Sun C, Liu S, Chai R, Huang W, Liu X, et al. Bioorganic fertilizer enhances soil suppressive capacity against bacterial wilt of tomato. PloS one. 2017; 10(4): e0121304. 10.1371/journal.pone.0121304 PMC4382293 25830639

[15] Ding C, Shen Q, Zhang R, Chen W, et al. Evaluation of rhizosphere bacteria and derived bio-organic fertilizers as potential biocontrol agents against bacterial wilt (Ralstonia solanacearum) of potato. Plant and Soil. 2013; 366(1/2): 453–466.

[16] Wang J, Zhu Z, Qian X, Wang G. Effects of reducing chemical fertilizer combined with application of different organic fertilizers on soil bacterial community structure during rice season. Soils. 2021; 53(05): 983–990. Chinese.

[17] Zou T, Kang Y, Wang B, Avila JM, You J, Zhu MJ, et al. Raspberry supplementation reduces lipid accumulation and improves insulin sensitivity in skeletal muscle of mice fed a high-fat diet. Journal of Functional Foods. 2019; 63(C): 103572–103572.

[18] Luo T, Chen S, Zhang H, Jia S, Wang J, et al. Phytochemical composition and potential biological activities assessment of raspberry leaf extracts from nine different raspberry species and raspberry leaf tea. Journal of Berry Research. 2020; 10(2): 295–309.

[19] Zhang L, Qiu J, Wang X, Wang X, Chang F, Yuan X, et al. Effects of exogenous melatonin on physiological characteristics of leaves under low-temperature stress in red raspberry. Journal of Fruit Science. 2022; 39(03): 416–425. Chinese.

[20] Institute of Soil Science, Chinese Academy of Sciences. Soil physical and chemical analysis. Shanghai Scientific and Technical Publishers; 1977. P. 142–231.

[21] Martin Marcel. Cutadapt removes adapter sequences from high-throughput sequencing reads. EMBnet. 2011; 17(1): pp-10.

[22] Callahan BJ, Mcmurdie PJ, Rosen MJ, Han AW, Johnson AJ, Holmes SP. Dada2: high-resolution sample inference from illumina amplicon data. Nature Methods. 2016; 13(7): 581–583. doi: 10.1038/nmeth.3869 27214047 PMC4927377

[23] Lozupone C, Knight R. UniFrac: a new phylogenetic method for comparing microbial communities. Applied and Environmental Microbiology. 2005; 71(12): 8228–8235. doi: 10.1128/AEM.71.12.8228-8235.2005 16332807 PMC1317376

[24] Lozupone CA, Hamady M, Kelley ST, Knight R. Quantitative and qualitative beta diversity measures lead to different insights into factors that structure microbial communities. Appl Environ Microbiol. 2007; 73: 1576–1585. doi: 10.1128/AEM.01996-06 17220268 PMC1828774

[25] Segata N, Izard J, Waldron L, Gevers D, Miropolsky L, Garrett WS, et al. Metagenomic biomarker discovery and explanation. Genome Biol. 2011; 12. doi: 10.1186/gb-2011-12-6-r60 21702898 PMC3218848

[26] Ramirez KS, Craine JM, Fierer N. Consistent effects of nitrogen amendments on soil microbial communities and processes across biomes. Global Change Biology. 2012; 18(6): 1918–1927.

[27] Zeng J, Liu X, Song L, Lin X, Zhang H, Shen C, et al. Nitrogen fertilization directly affects soil bacterial diversity and indirectly affects bacterial community composition. Soil Biology and Biochemistry. 2016; 92: 41–49.

[28] Ye S, Zheng C, Zhang Y, Liu X. Effects of reduced chemical nitrogen input combined with organic fertilizer application on the productivity of winter wheat and summer maize rotation and soil properties in central Henan Province. Chinese Journal of EcoAgriculture, 2022; 30(6): 900−912. Chinese.

[29] Yang L, Wang Y, Han W, Ma L, Yang G, Han Y, et al. Effects of reducing nitrogen fertilizer and applying organic fertilizer on apple yield and quality and soil biological characteristic. Journal of Agro-Environment Science. 2021; 40(03): 631–639. Chinese.

[30] Zhang L, Huang J, Gao J, Cao W, Gao P, Yang Z. Effects of long-term green manure and reducing nitrogen applications on rice yield and soil nutrient content. Transactions of the Chinese Society of Agricultural Engineering. 2020; 36(05): 106–112. Chinese.

[31] He H, Zhang Y, Wei C, Li J. Effects of different organic substitution reducing fertilizer patterns on maize growth and soil fertility. Journal of Soil and Water Conservation. 2019; 33(05): 281–287. Chinese.

[32] Wang S, Wang J, Zhou Y, Huang Y, Tang X. Isolation, classification, and growth-promoting effects of Pantoea sp. YSD J2 from the aboveground leaves of Cyperus esculentus L. var. sativus. Current Microbiology. 2022; 79(2): 66. doi: 10.1007/s00284-021-02755-8 35059843

[33] Zhou Y, Bai Y, Yue T, Li Q, Huang Y, Jiang W, et al. Research progress on the growth-promoting characteristics of plant growth-promoting rhizobacteria. Microbiology China. 2023; 50(02): 644–666. Chinese.

[34] Cheng H, Liu S, Yang W, Liang G. Structure and diversity of bacterial community in rhizosphere soil of four dominant species along the bank of the lower reaches of Yarlung Zangbo River. Acta Ecologica Sinica. 2022; 42(04): 1527–1537. Chinese.

[35] Sun H, Li X, Jin L, Zhao Y, Li C, Zhang J, et al. Changes in soil bacterial community diversity in degraded patches of alpine meadow in the source area of the Yellow River. Environmental Science. 2022;43(09):4662–4673. Chinese. doi: 10.13227/j.hjkx.202112106 36096607

[36] Zhang M, Zhang W, Bai SH, Niu Y, Hu D, Ji H, et al. Minor increases in Phyllostachys edulis (Moso bamboo) biomass despite evident alterations of soil bacterial community structure after phosphorus fertilization alone: Based on field studies at different altitudes. Forest Ecology and Management. 2019; 451(C): 117561–117561.

[37] Ren M, Zhang Z, Wang X, Zhou Z, Chen D, Zeng H, et al. Diversity and Contributions to Nitrogen Cycling and Carbon Fixation of Soil Salinity Shaped Microbial Communities in Tarim Basin. Frontiers in Microbiology. 2018; 9: 431. doi: 10.3389/fmicb.2018.00431 29593680 PMC5855357

[38] Radhey SG, Amy M. Phylogenomics and signature proteins for the alpha Proteobacteria and its main groups. BMC Microbiology. 2007; 7(1): 1–20. 18045498 10.1186/1471-2180-7-106 PMC2241609

[39] Du S, Yu M, Liu F, Xiao L, Zhang H, Tao K, et al. Effect of facility management regimes on soil bacterial diversity and community structure. Chinese Journal of Eco-Agriculture. 2017; 25(11): 1615–1625. Chinese.

[40] Campbell BJ, Polson SW, Hanson TE, Mack MC, Schuur EAG. The effect of nutrient deposition on bacterial communities in Arctic tundra soil. Environmental microbiology. 2010; 12(7): 1842–54. doi: 10.1111/j.1462-2920.2010.02189.x 20236166

[41] Li C, Yang J, Wang X, Wang E, Li B, He R, et al. Removal of nitrogen by heterotrophic nitrification–aerobic denitrification of a phosphate accumulating bacterium Pseudomonas stutzeri YG-24. Bioresource Technology. 2015; 182: 18–25. doi: 10.1016/j.biortech.2015.01.100 25668754

[42] Yu Y, Wang H, Liu J, Wang Q, Shen T, Guo W, et al. Shifts in microbial community function and structure along the successional gradient of coastal wetlands in Yellow River Estuary. European Journal of Soil Biology. 2012; 49:12–21.

[43] Wang X, Ma K, Fu Y, An Y, Wang Z. Effects of no-tillage mulching and bioorganic fertilizer on soil bacterial community in winter wheat. Acta Ecologica Sinica. 2020; 40(19): 7030–7043. Chinese.

[44] An X, Jiang S, Xie C, Xu Y, Dong C, Shen Q. Effects of reducing chemical fertilizers combined with organic fertilizers on soil microbial community in litchi orchards. Chinese Journal of Applied Ecology. 2022; 33(04): 1099–1108. Chinese. 35543065 10.13287/j.1001-9332.202204.031

[45] Arah JRM, Kirk GJD. Modeling rice plant-mediated methane emission. Nutr Cycl Agroecosyst, 2000; 58:221–230.

[46] Liu D, Qiao Y, Li S, Cheng Y, Zhang Z, Li F, et al. Effects of long-term fertilizer application on bacterial diversity in a yellow brown soil. Journal of Plant Nutrition and Fertilizers. 2021; 27(05): 760–767. Chinese.

[47] Shang L, Wan L, Li X. Effects of organic fertilizer on soil bacterial community diversity in leymus chinensis steppe. Scientia Agricultura Sinica. 2020; 53(13): 2614–2624. Chinese. 10.1371/journal.pone.0240559 PMC7561123 33057441

[48] Cai J, Zhang J, Yu S, Lin H, Li K, Chen S, et al. Effect of fertilization on bacterial diversity and community structure characteristics in cassava rhizospheric soil. Journal of Fujian Agriculture and Forestry University. 2022; 51(01): 15–20. Chinese.

[49] Zhao J, Wang F, Meng C, Jin S, Peng M. Effects of bio-organic fertilizer on potato yield and abundances of soil nitrogen-cycling microbes. Transactions of the Chinese Society for Agricultural Machinery. 2022; 1–13. Epub 2022 Feb 25. Chinese.

[50] Li W, Wu M, Liu M, Jiang C, Chen X, Kuzyakov Y, et al. Responses of Soil Enzyme Activities and Microbial Community Composition to Moisture Regimes in Paddy Soils Under Long-Term Fertilization Practices. Pedosphere. 2018; 28(2): 323–331.

[51] Wyk DAB, Adeleke R, Rhode OHJ, Bezuidenhout CC, Mienie C. Ecological guild and enzyme activities of rhizosphere soil microbial communities associated with Bt-maize cultivation under field conditions in North West Province of South Africa. Journal of basic microbiology. 2017; 57(9): 781–792. doi: 10.1002/jobm.201700043 28731210

[52] Batista ER, Carneiro JJ, Pinto FA, Santos JV, Carneiro MAC. Environmental drivers of shifts on microbial traits in sites disturbed by a large-scale tailing dam collapse. Science of the Total Environment. 2020; 738: 139453. doi: 10.1016/j.scitotenv.2020.139453 32531582

[53] Liu W, Wang Q, Wang B, Wang X, Franks AE, Teng Y, et al. Changes in the abundance and structure of bacterial communities under long-term fertilization treatments in a peanut monocropping system. Plant and Soil. 2015; 395: 415–427.

[54] Yao Q, Liu J, Yu Z, Li Y, Jin J, Liu X, et al. Changes of bacterial community compositions after three years of biochar application in a black soil of northeast China. Applied Soil Ecology; 2017, 113:11–21.

[55] Zhou L, Li H, Zhang Y, Han S, Xu H. *Sphingomonas* from petroleum-contaminated soils in Shenfu, China and their PAHs degradation abilities. Brazilian Journal of Microbiology. 2016; 47(2):271–278. 26991271 10.1016/j.bjm.2016.01.001 PMC4874584

[56] Guo J, Chen Y, Lu P, Liu M, Sun P, Zhang Z, et al. Roles of endophytic bacteria in Suaeda salsa grown in coastal wetlands: Plant growth characteristics and salt tolerance mechanisms. Environmental Pollution. 2021; 287:117641–117641. doi: 10.1016/j.envpol.2021.117641 34426384

[57] Griffiyhs BS, Philippot L. Insights into the resistance and resilience of the soil microbial community. FEMS Microbiology Reviews. 2013; 37(2): 112–129. doi: 10.1111/j.1574-6976.2012.00343.x 22568555

[58] Zhang Q, Zhang W. Microbial flora analysis for the degradation of beta-cypermethrin. Environmental Science and Pollution Research. 2017; 24(7): 6554–6562. doi: 10.1007/s11356-017-8370-5 28074371

[59] Liu Q, Wang S, Li K, Qian J, Guo Y, Liu Z, et al. Responses of soil bacterial and fungal communities to the long-term monoculture of grapevine. Applied Microbiology and Biotechnology. 2021; 105(18):7035–7050. doi: 10.1007/s00253-021-11542-1 34477939

[60] Wang L, Wang LA, Zhan X, Huang Y, Wang J, Wang X. Response mechanism of microbial community to the environmental stress caused by the different mercury concentration in soils. Ecotoxicology and Environmental Safety. 2020; 188(C):109906. 31708226 10.1016/j.ecoenv.2019.109906

[61] Ntougias S, Melidis P, Navrozidou E, Tzegkas F. Diversity and efficiency of anthracene-degrading bacteria isolated from a denitrifying activated sludge system treating municipal wastewater. International Biodeterioration & Biodegradation. 2015; 97:151–158.

[62] Sun QQ, Zheng YM, Yu TY, Wu Y, Yang JS, Wu ZF, et al. Responses of soil diazotrophic diversity and community composition of nodulating and non-nodulating peanuts (Arachis hypogaea L.) to nitrogen fertilization. Acta Agronomica Sinica. 2022; 48(10): 2575–2587. Chinese.

[63] Oliveira A, Pampulha ME., Neto MM, Almeida AC. Mercury tolerant diazotrophic bacteria in a long-term contaminated soil. Geoderma. 2010; 154: 359–363.

[64] Karthikeyan S, Rodriguez-R LM, Heritier-Robbins P, Kim M, Overholt WA, Gaby JC, et al. "Candidatus Macondimonas diazotrophica", a novel gammaproteobacterial genus dominating crude-oil-contaminated coastal sediments. The ISME Journal. 2019; 13(8): 2129–2134. doi: 10.1038/s41396-019-0400-5 30952995 PMC6776044

[65] Naseri M, Barabadi A, Barabady J. Bioremediation treatment of hydrocarbon-contaminated Arctic soils: influencing parameters. Environmental Science and Pollution Research. 2014; 21(19): 11250–11265. doi: 10.1007/s11356-014-3122-2 24903252

[66] Yang Y, Wang Z, Zeng Z. Effects of long-term different fertilization and irrigation managements on soil bacterial abundance, diversity and composition. Scientia Agricultura Sinica. 2018; 51(02): 290–301. Chinese.

[67] Zhou J, Guan D, Zhou B, Zhao B, Ma M, Qin J, et al. Influence of 34-years of fertilization on bacterial communities in an intensively cultivated black soil in northeast China. Soil Biology and Biochemistry. 2015; 90: 42–51.

[68] Yang Y, Huang X, Zhu H, Li Y, Zhang S, Zhang Y, et al. Bacterial community structure and composition under long-term combined application of organic and inorganic fertilizers in a yellow paddy soil. Journal of Plant Nutrition and Fertilizers. 2022; 28(06):984–992. Chinese.

[69] Liu L, Li T, Wei X, Jiang B, Ping F. Effects of a nutrient additive on the density of functional bacteria and the microbial community structure of bioorganic fertilizer. Bioresource Technology. 2014; 172: 328–334. doi: 10.1016/j.biortech.2014.08.125 25280041

[70] Wu Y, Zhao C, Farmer, J, Sun J. Effects of bio-organic fertilizer on pepper growth and Fusarium wilt biocontrol. Scientia Horticulturae. 2015; 193: 114–120.

[71] Li G, Li P, Wu M, Li Z. Effects of chemical fertilizer combined with organic manure on peanut rhizosphere bacterial community structure and co-occurrence network. Soils. 2022; 54(03): 498–507. Chinese.

[72] Shi R, Wang S, Xiong B, Gu H, Wang H, Ji C, et al. Application of Bioorganic Fertilizer on Panax notoginseng Improves Plant Growth by Altering the Rhizosphere Microbiome Structure and Metabolism. Microorganisms. 2022; 10(2): 275–275. doi: 10.3390/microorganisms10020275 35208730 PMC8879206

[73] Fisk LM, Barton L, Jones DL, Glanville HC, Murphy DV. Root exudate carbon mitigates nitrogen loss in a semi-arid soil. Soil Biology and Biochemistry. 2015; 88: 380–389.

[74] Huang N, Wang W, Yao Y, Zhu F, Wang W, Chang X. The influence of different concentrations of bio-organic fertilizer on cucumber Fusarium wilt and soil microflora alterations. PLoS ONE. 2017; 12(2): e0171490. doi: 10.1371/journal.pone.0171490 28166302 PMC5293216

[75] Jin N, Jin L, Wang S, Li J, Liu F, Liu Z, et al. Reduced Chemical Fertilizer Combined With Bio-Organic Fertilizer Affects the Soil Microbial Community and Yield and Quality of Lettuce. Frontiers in Microbiology. 2022; 13: 863325–863325. doi: 10.3389/fmicb.2022.863325 35531292 PMC9069001

[76] Xiao X, Li J, Lyu J, Feng Z, Zhang G, Yang H, et al. Chemical fertilizer reduction combined with bio-organic fertilizers increases cauliflower yield via regulation of soil biochemical properties and bacterial communities in Northwest China. Frontiers in Microbiology. 2022; 13: 922149–922149. doi: 10.3389/fmicb.2022.922149 35966650 PMC9363920

